# Supplementary material for: PIQLE: protein–protein interface quality estimation by deep graph learning of multimeric interaction geometries
Source: Bioinform Adv. 2023 Jun 2;3(1):vbad070. doi: 10.1093/bioadv/vbad070 (PMC10281963; doi:10.1093/bioadv/vbad070)

Supplementary Information
for

PIQLE: protein-protein interface quality estimation by deep graph learning of multimeric interaction geometries

Md Hossain Shuvo[1], Mohimenul Karim[1], Rahmatullah Roche[1], Debswapna Bhattacharya[1,*]

[1]Department of Computer Science, Virginia Tech, Blacksburg, VA 24073, USA.

*To whom correspondence should be addressed. Phone: (540) 231-2865. E-mail: dbhattacharya@vt.edu

Supplementary Table S1. Categorization of several node features adopted in PIQLE.

	Properties	Category
5-state amino acid residue types	ALA VAL LEU ILE PRO PHE MET TRP	Nonpolar
	GLY SER THR CYS TYR ASN GLN	Polar
	LYS ARG HIS	Positively charged
	ASP GLU	Negatively charged
	Any other residues	Neutral/None
3-state secondary structure types	G, H, I	H
	E, B	E
	Other	C
2-state solvent accessibility types	RSA (ACC / Max. SA) < 25%	B
	RSA (ACC / Max. SA) > 25%	E

Supplementary Table S2. Hyperparameter selection for graph attention network (GAT), Graph Transformer Network (GTN), and Graph Convolutional Network (GCN) on the independent ZDOCK validation dataset in terms of Spearman correlations coefficient (ρ) between the estimated protein-protein interface quality scores and their corresponding DockQ scores.

**Hyperparameter**		**GAT**	**GTN**	**GCN**
**# Layers**	**# Heads**	**Spearman ρ**	**Spearman ρ**	**Spearman ρ**
**4**	2	0.168	0.116	0.219
	**4**	**0.367**	0.048	
	6	0.199	0.042	
	8	0.266	0.067	
6	2	0.155	0.042	**0.264**
	4	0.178	0.015	
	6	0.300	0.074	
	8	0.288	0.122	
8	2	0.222	0.039	0.171
	4	0.280	0.085	
	6	0.325	0.041	
	8	0.289	0.053	

10	2	0.200	0.071	0.214
	4	0.189	0.056	
	6	0.172	0.108	
	8	0.185	0.040	
12	2	0.173	0.099	0.209
	4	0.218	0.121	
	6	0.230	0.070	
	8	0.227	0.044	
14	2	0.220	0.127	0.193
	4	0.199	0.036	
	6	0.202	0.095	
	8	0.235	0.056	
16	2	0.139	**0.145**	0.240
	4	0.238	0.041	
	6	-0.020	0.037	
	8	0.225	0.042	

Note: Graph attention network with the hyperparameters in bold yield the highest Spearman ρ

Supplementary Table S3. Pairwise sequence identity between training, testing, and validation datasets.

Datasets	Dockground v1[a]	Dockground v2[b]	ZDOCK[c]	HAF2[d]
**Dockground v1**		0.183	0.192	0.162
**Dockground v2**	0.183		0.179	0.152
**ZDOCK**	0.192	0.179		0.133
**HAF2**	0.162	0.152	0.133	

[a, d]Testing datasets
[b]Training dataset
[c]Validation dataset
[d]Heterodimer-AF2 dataset

Supplementary Figure S1. Ranking complex structural models with acceptable quality (i.e., DockQ scores ranging between 0.23 and 0.49) for PIQLE and the competing methods in terms of success rate on (**a**) Dockground v1 dataset, (**b**) HAF2 dataset and hit rate on (**c**) Dockground v1 dataset, (**d**) HAF2 dataset based on top-1, top-5, top-10, top-15, top-20, top-25, and top-30 models.

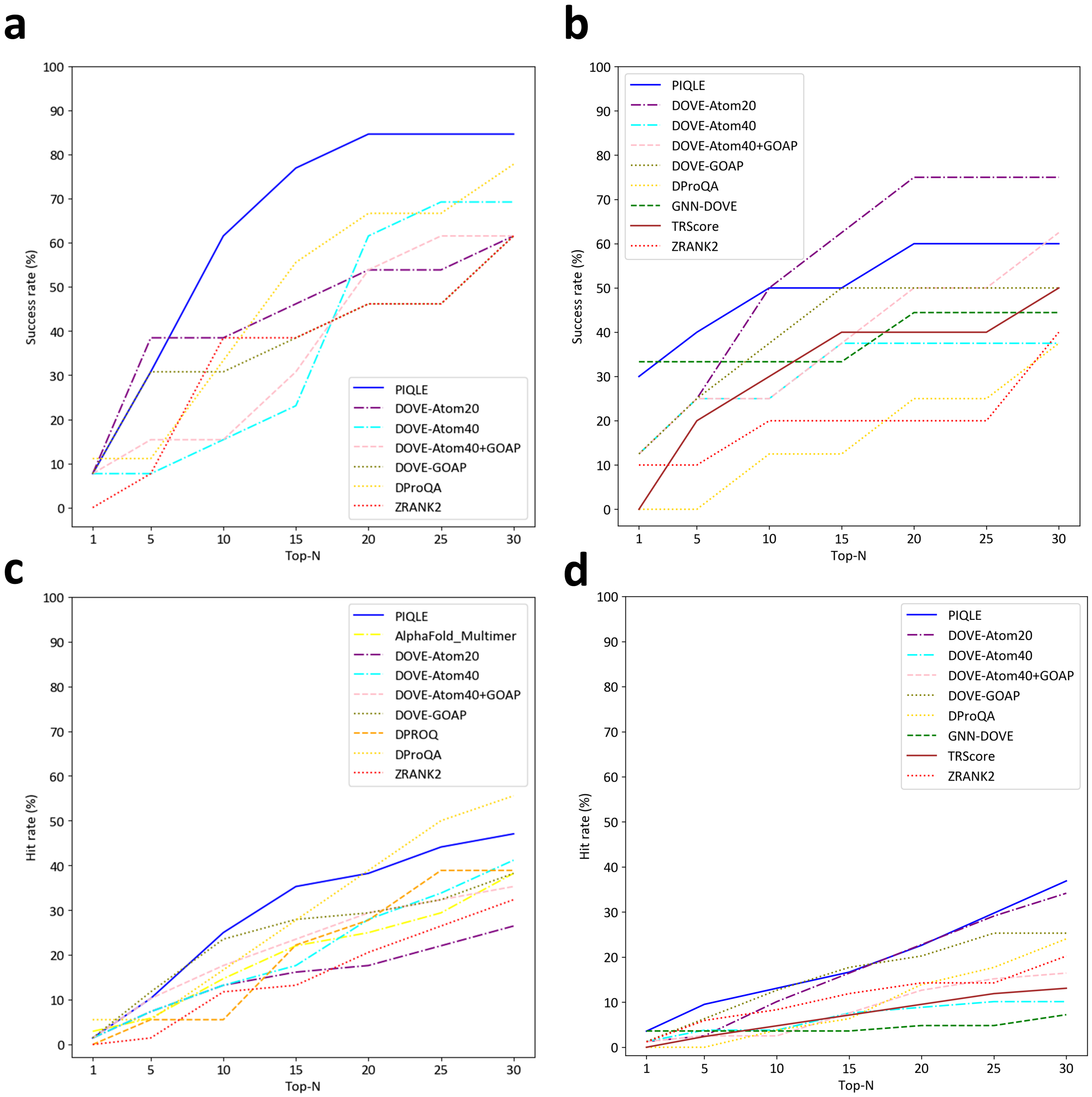

Supplementary Figure S2. Ranking complex structural models with medium quality (i.e., DockQ scores ranging between 0.49 and 0.80) for PIQLE and the competing methods in terms of success rate on (**a**) Dockground v1 dataset, (**b**) HAF2 dataset and hit rate on (**c**) Dockground v1 dataset, (**d**) HAF2 dataset based on top-1, top-5, top-10, top-15, top-20, top-25, and top-30 models.

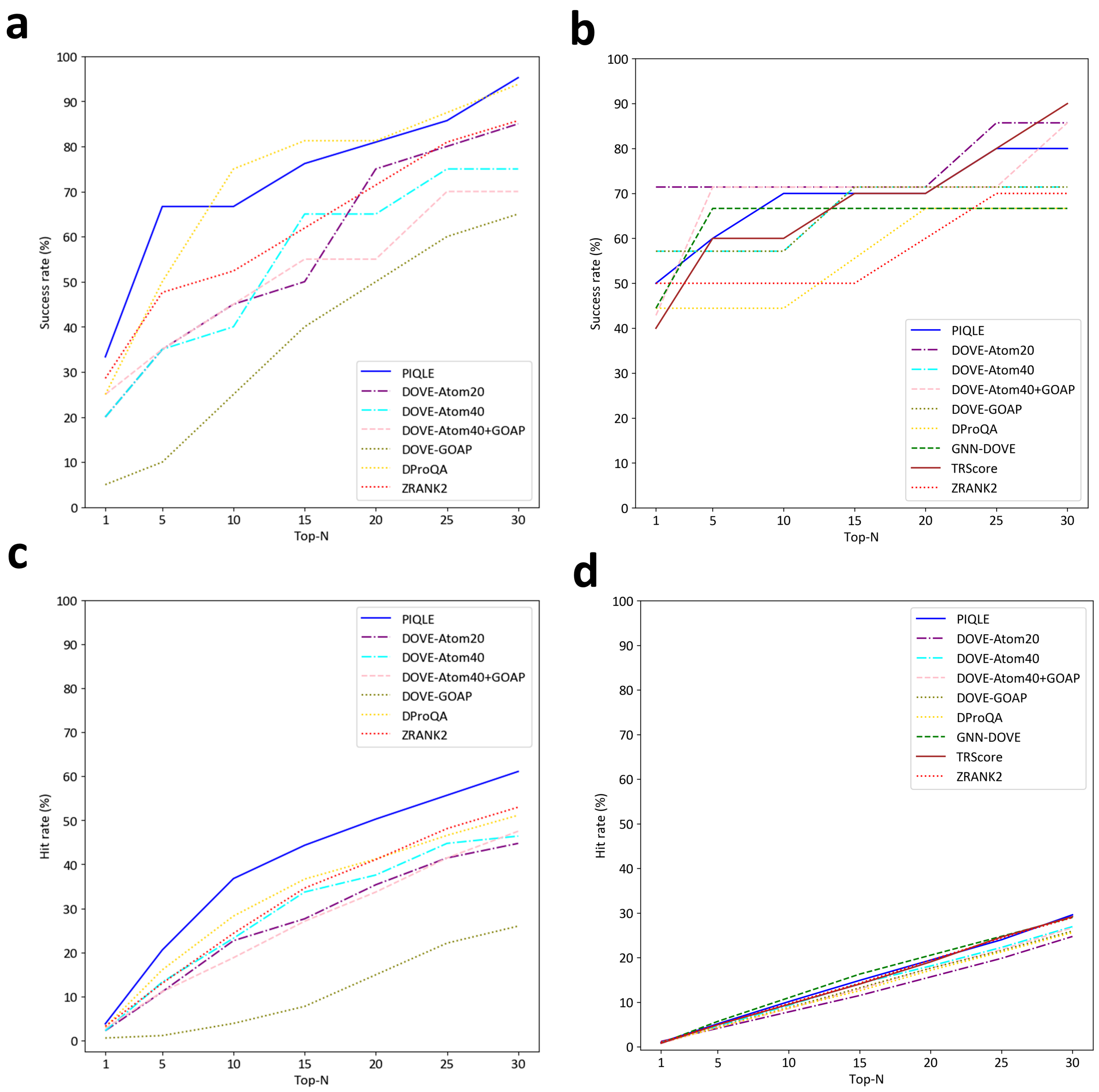

Supplementary Figure S3. Ranking complex structural models with high quality (i.e., DockQ scores more than or equal to 0.80) for PIQLE and the competing methods in terms of success rate on (**a**) Dockground v1 dataset, (**b**) HAF2 dataset and hit rate on (**c**) Dockground v1 dataset, (**d**) HAF2 dataset based on top-1, top-5, top-10, top-15, top-20, top-25, and top-30 models.

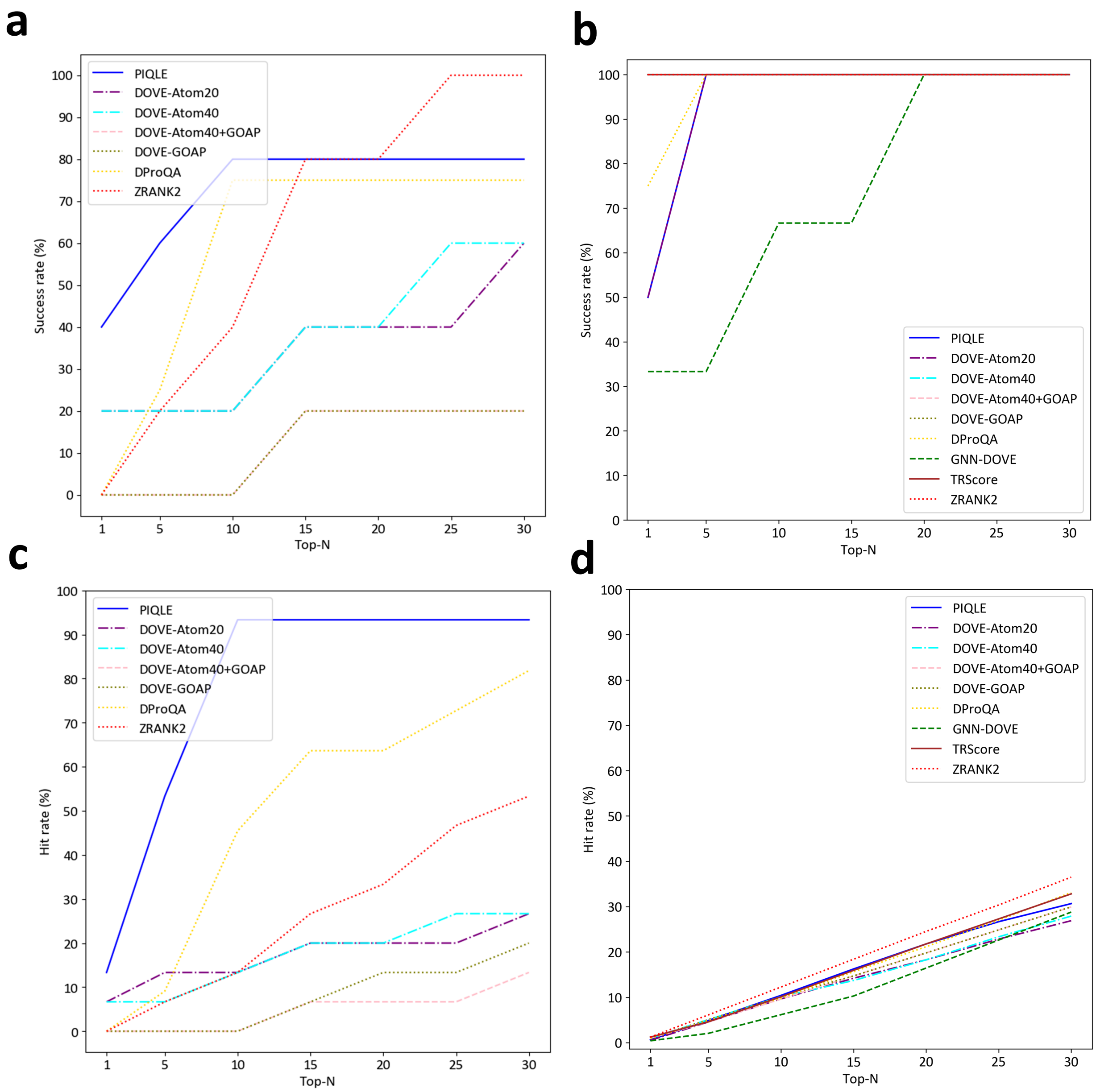

Supplement: vbad070_Supplementary_Data [file vbad070_supplementary_data.pdf]
